# Status and factors related to hemoglobin concentration of people with vs. without disability—using nationwide claims check-up database

**DOI:** 10.3389/fnut.2025.1519098

**Published:** 2025-03-19

**Authors:** Seyune Lee, Young-Il Jung, Hyejung Yoon, Se-Youn Jung, Boyoung Jeon, In-Hwan Oh, Su Jin Jeong

**Affiliations:** ^1^Department of Environmental Health, Korea National Open University, Seoul, Republic of Korea; ^2^Prime College, Korea National Open University, Seoul, Republic of Korea; ^3^Department of Health and Medical Information, Myongji College, Seoul, Republic of Korea; ^4^Department of Preventive Medicine, College of Medicine, Kyung Hee University, Seoul, Republic of Korea; ^5^Statistics Support Part, Clinical Research Institute, Kyung Hee University Medical Center, Seoul, Republic of Korea

**Keywords:** persons with disability, hemoglobin, Anemia, PSM, nutritional inequality, claims data analysis

## Abstract

**Background:**

Blood hemoglobin level is a key indicator of organ function and health status throughout the life course. As hemoglobin-related health problems are gaining attention, many studies on factors related to hemoglobin concentration are being conducted, yet few researches have been conducted targeting persons with disabilities. Furthermore, researches that consider the association between blood hemoglobin and the regional level health welfare resources are rare. This study aimed to explore the factors related to blood hemoglobin concentration among people with and without disability, and to contribute to the development of future nutritional policies and projects for persons with disabilities.

**Methods:**

A health insurance claims database with check-ups provided by the National Health Insurance Services (NHIS) was used. One-to-three propensity score matching was conducted between participants with and without disabilities. In addition to the individual clinical and health behavioral aspects based on the claims data, regional-level data of healthcare and social welfare resources was also collected, and multi-level analysis was conducted to identify factors associated with low blood hemoglobin level.

**Results:**

A total of 1,697 participants with disability and 5,091 without disabilities were yielded. Disability was significantly associated with lower hemoglobin level, even after propensity score matching. Sex, BMI, health behaviors, and clinical indicators were significantly associated with the blood hemoglobin level. Furthermore, region-level welfare budget was a significant factor among persons with disability.

**Conclusion:**

Our findings confirmed the significant association between disability and lower hemoglobin level. Regional health and welfare resources, as well as individual characteristics should be considered in implementation of further nutrition and health policies for persons with disabilities. Further studies are needed to understand of health outcomes of low hemoglobin level.

## 1 Introduction

Anemia, defined as the low level of blood hemoglobin, is a common nutrition and health problem throughout the world (1). The World Health Organization (WHO) defined anemia as ‘a condition in which the number of red blood cells is insufficient to meet the body’s physiological needs’ (2). Although anemia itself is clearly related to poor nutritional status, it is also associated with various diseases and other health conditions (3). There are various types and causes of anemia, and it is necessary to consider the medical history of gastrointestinal bleeding, liver or kidney disease, and infections to diagnose anemia. Blood hemoglobin level, which is a key indicator of organ function and health status is the most common and basic approach of confirming anemia (4, 5).

In fact, the blood level of hemoglobin itself is related to significant health effects such as anemia, osteoporosis, hemodialysis (3). The low blood level of hemoglobin is also related to the occurrence of adverse events such as certain type of cancers, and other clinical conditions in overall population (6–8). It is also recognized a risk factor of allergic diseases and even postpartum depression in women (8, 9). Furthermore, studies with older population report that hemoglobin level is also related to reduce cognitive function and physical performance, low bone mass, eventually leading to disability (10, 11).

Anemia, recognized as a challenging public health problem among vulnerable populations, previous studies have focused on the blood hemoglobin levels of pregnant women, infants, adolescent girls, and older populations (1, 10, 12–14). However, there has been a dearth of in-depth research targeting persons with disabilities, a significant group with health vulnerabilities. In fact, approximately 15% of the world population experience disability (15), and as of 2023, there are 2.647 million were registered as ‘persons with disabilities’ in South Korea. This number has been increasing over the past 10 years, and is expected to continue to increase especially with population aging.

Persons with disabilities tend to be more sensitive to chronic diseases compared to non-disabled people due to barriers that they face - unavailability of health screening, limitation in providers and information, etc. (16). According to previous studies, persons with disabilities have shown to have a higher prevalence of chronic diseases, more severe complications, and shorter life expectancy compared to non-disabled people (17, 18). Specifically, the proportion of those with chronic diseases among the disabled is approximately 85%, which is significantly higher compared to the overall population (19). Marrocco also found that persons with disabilities are less likely to receive preventive health care services, and have fewer opportunities to stay healthy (20).

In this situation, actions and movements to remove barriers and to improve access to health services of persons with disabilities have been spreading worldwide (21). In particular, the importance of prevention and management before the onset of a disease is being highlighted, and accordingly, there is a need to understand individual and regional factors affecting chronic diseases (22). As part of this, various food and nutritional factors and status among persons with disabilities were explored (23). Studies found a distinct relationship between inadequate nutritional intake and disability, which constitute a large share of the overall health problems (24).

In terms of blood hemoglobin levels, specific disability groups such as those with physical disabilities or cerebral palsy may have abnormal hemoglobin levels due to problems with blood circulations (11, 25). Conversely, research have also reported that low hemoglobin concentration affects the ability to perform daily living activities, physical ability, and the occurrence of disabilities (26). Therefore, the management of hemoglobin levels among persons with disabilities can be regarded as an important policy agenda (27).

According to previous studies which performed multi-level analysis to explore community-level related factors in the occurrence of anemia found that the socioeconomic level of the region, accessibility to healthy foods and health care services, provision of nutrition-related policies and projects, and even traditions and culture can affect the individuals’ blood hemoglobin level (3). In fact, policies related to support and care for persons with disabilities, including community health and medical resources, play an important role in preventing the occurrence of diseases and health promotion (28).

As mentioned above, while studies on hemoglobin concentrations are being conducted, research targeting persons with disabilities is still limited. Furthermore, to our knowledge, no study has analyzed the impact of the regional or community level variables on hemoglobin levels among persons with disabilities. The aim of this study is to explore the factors related to blood hemoglobin concentration of people with and without disability, and to contribute to the development of future nutritional policies and projects for persons with disabilities. In particular, this study seeks to address the association between individual blood hemoglobin level and regional-level healthcare and social welfare resources.

## 2 Materials and methods

### 2.1 Data source and study design

This study is a secondary data analysis using nationwide population-based data. Data was retrieved from the National Health Insurance Services (NHIS) claims database combined with the national health check-up data. The NHIS is a single-payer health care system in Korea and covers majority of South Koreans. Additionally, under the National Health Insurance, those over the age of 40 are expected to undergo a health check-up including blood tests, self-reported questionnaires, and further examinations at least every 2 years. The subject group of this study include those who have been newly assigned a disability code as of 2010–2019. The control group is defined as those who had never been given a disability grade in the past, and they are pre-matched 1 on1 with the subject group based on gender and age. In this study, those who have received health check-ups in either 2018 or 2019 were screened for eligibility. If a person was screened in both 2018 and 2019, the latter result was adopted.

**Figure 1:**
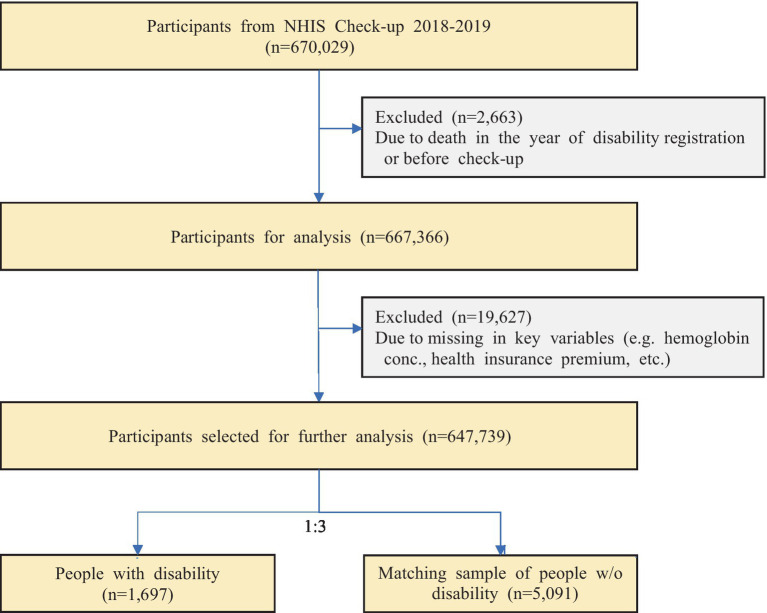
Flowchart of participant selection process. NHIS, National Health Insurance Services.

Of the sample of 670,029 participants, those who died in the year of disability registration or before the health check-up, and those with missing in key dependent or independent variables (e.g., hemoglobin conc., health insurance premium, etc.) were excluded which yielded a remaining sample of 647,739. In addition, people registered as disabled due to internal or liver disorders were not included in the final analysis as bias in their blood hemoglobin levels was expected. People with facial disorder was also excluded due to the differences in nature compared to other disabilities. The specific process of selecting the study participants included in the analysis was presented in Figure 1.

### 2.2 Definition of variables

The dependent variable of the study, blood hemoglobin concentration, was obtained from the first venous blood test tube during health check-ups and was expressed in grams per deciliter (g/dL). According to the NHIS health check-up guidelines, individuals with blood hemoglobin concentrations less than 12.0 g/dL in men and less than 10.0 g/dL in women were defined as ‘suspected disease’, and were categorized as having hemoglobin deficiency (abnormal) in this analysis (29). During data processing, individuals with hemoglobin concentrations of zero or higher than 25.0 g/dL were treated as outliers and excluded.

Regarding covariates, comorbidities were calculated using the updated Charlson Comorbidity Index (CCI) (30), which is widely used in healthcare research using claims data, and recategorized as 0, 1, 2 or above. Body Mass Index (BMI) was calculated using height and weight, and categorized according to the Asia Pacific classification (31). Under this classification, individuals with BMI < 18.5 kg/m2 were defined as underweight, 18.5–22.9 kg/m2 as normal weight, 23.0–24.9 kg/m2 as overweight, and ≥ 25.0 as obese. Systolic and diastolic blood pressure measurements were taken, and values considered outliers were excluded (systolic: less than 60 or higher than 400; diastolic: less than 30 or higher than 250). Fasting blood glucose was measured after an 8 to 12-h fasting period. To assess kidney function, serum creatinine concentration and urine protein were collected (excluding urine protein values of 0 or higher than 6). For liver function assessment, aspartate aminotransferase (AST), which measures enzymes indicating liver cell damage was measured. Additionally, *γ*-glutamyl transpeptidase (GTP), which indicates fatty liver, was measured. Smoking and drinking behaviors, along with frequency of physical activity, were collected through self-reported questionnaires.

Several regional-level variables indicating healthcare and welfare resources were included in the analysis. The ‘number of doctors per 1,000 people’ is defined as the number of doctors, traditional medicine doctors, and dentists currently working in medical institutions per 1,000 people. This indicator is commonly used to determine appropriate health workforce levels, with higher numbers indicating better medical welfare (32). The ‘number of social welfare facilities per 100,000 people’ was also included. These facilities serve older populations, women, children, persons with disabilities, and homeless, individuals, serving as an indicator of welfare consideration for vulnerable groups (33). Welfare facilities provide diverse services, from care and social service delivery to healthcare service coordination, and are particularly important for persons with disabilities during their initial health assessments (34). The ‘social welfare budget ratio’ represents the proportion of social welfare and health spending in a local government’s total annual budget. Higher values are interpreted as having more positive effects on population quality of life (35). These social welfare budgets cover basic pensions, activity assistance benefits, transportation fees, and healthcare benefits for the low-income groups. Therefore, regional variations can impact healthcare accessibility for persons with disabilities (36, 37).

These regional level variables (at the city/county/district level) collected in 2018 were provided by Statistics Korea (The description of the regional level variables is available in the Supplementary Table 1).

### 2.3 Statistical analysis

Propensity scores for each participant were calculated using logistic regression, adjusted for age, sex, health insurance premium (as a proxy variable for income status), and Charlson Comorbidity Index (CCI). Propensity score matching is a statistical methodology that makes two different groups as homogeneous as possible, and is widely used in observational studies using healthcare big data (38). Participants with and without disabilities were matched in a 1:3 ratio, yielding a total of 6,788 subjects for further analysis.

Using this matched sample, associated factors of blood hemoglobin levels were examined. T-tests were used to compare continuous variables, and chi-square tests were used for categorical variables. To examine the variation in regional-level data, the quartiles of each variable were calculated, and the mean and standard deviation of each quartile were presented.

Additionally, multiple logistic regression analysis was performed to identify factors associated with low hemoglobin concentration in people with and without disabilities. For further analysis, multi-level analysis incorporating both individual, and regional level was conducted. To confirm model fit, log likelihood and AIC (Akaike Information Criterion) values of each model were compared. AIC is often used to measure model adequacy in multiple modeling (39). All data management and analysis were performed using SAS Enterprise Guide 7.4.

## 3 Results

General characteristics of participants with and without disability, before and after matching are shown in Table 1. Initially, the sample comprised 1,697 persons with disability and 646,042 without disabilities. After 1:3 matching based on sex, age, health insurance premium, and CCI, the sample included 1,697 persons with disabilities and 5,091 without disabilities. Before matching, people without disabilities showed a higher proportion of individuals in their 40s and 50s and a lower proportion in their 70s or older. After matching, there was no statistical difference in age distribution between the two groups. Regarding health insurance premiums, persons with disabilities initially showed higher proportion of medical aid recipients [0], while those without disabilities had a higher proportion in the highest premium category [4]. These differences disappeared after matching. Similarly, while people without disabilities initially showed a higher proportion of CCI scores of 0, the CCI distribution between groups showed no differences after matching (Distribution changes in propensity scores before and after matching are presented in the Supplementary Figure 1).

**Table 1:** General characteristics of participants before and after matching (1:3; %).

		Before matching			After matching		
		Disability	w/o disability		Disability	w/o disability	
		*n* = 1,697	*n* = 646,042		*n* = 1,697	*n* = 5,091	
Sex	Male	57.63	57.05	0.6269	57.63	59.10	0.2857
	Female	42.37	42.95		42.37	40.90	
Age	40	6.42	17.79	<0.0001	6.42	6.82	0.8221
	50	17.03	29.2		17.03	17.78	
	60	29.82	32.84		29.82	29.37	
	70+	46.73	20.16		46.73	46.04	
Health insurance premium	Medical aid	6.87	2.13	<0.0001	6.87	6.78	0.6630
	Q1	17.64	18.14		17.64	17.74	
	Q2	18.48	16.50		18.48	17.09	
	Q3	22.86	24.43		22.86	24.22	
	Q4	34.14	38.81		34.14	34.18	
CCI	0	51.09	73.04	<0.0001	51.09	49.62	0.5207
	1	16.85	10.86		16.85	16.93	
	2+	32.06	16.10		32.06	33.45	
BMI	18.5 <	11.48	2.76	<0.0001	11.48	4.22	<0.0001
	18.5–22.9	39.22	31.98		39.22	34.02	
	23.0–24.9	17.81	26.54		17.81	26.09	
	25 ≤	31.49	38.72		31.49	35.67	
Waist circumference	Mean (S.D)	83.31 (10.07)	83.61 (8.81)	0.1692	83.31 (10.07)	83.85 (9.02)	0.0514
Smoking	No	68.06	63.51	<0.0001	68.06	65.47	0.0100
	Yes	24.10	29.57		24.10	28.28	
	Ever	7.84	6.92		7.84	6.25	
Drinking	No	79.91	59.66	<0.0001	79.91	67.07	<0.0001
	2-3/mon.	4.60	11.10		4.60	7.74	
	1-2/wk.	8.19	17.69		8.19	13.89	
	3/wk.+	7.31	11.55		7.31	11.30	
Physical activity	No	66.65	74.80	<0.0001	66.65	49.91	<0.0001
	1-2/wk.	8.43	10.06		8.43	12.41	
	3/wk.+	24.93	15.14		24.93	37.67	
BP-diastolic	Mean (S.D)	75.22 (10.59)	76.48 (9.85)	<0.0001	75.22 (10.59)	75.99 (9.89)	0.0062
BP-systolic	Mean (S.D)	128.00 (17.65)	128.10 (15.30)	0.7154	128.00 (17.65)	129.70 (15.57)	0.0005
Fasting BG	Mean (S.D)	112.20 (40.29)	106.5 (26.94)	<0.0001	112.20 (40.29)	108.20 (28.92)	<0.0001
Creatinine	Mean (S.D)	1.10 (0.85)	0.90 (0.51)	<0.0001	1.10 (0.85)	0.95 (0.94)	<0.0001
Urine protein	Mean (S.D)	1.32 (0.88)	1.13 (0.52)	<0.0001	1.32 (0.88)	1.15 (0.58)	<0.0001
γ-GTP	Mean (S.D)	49.64 (101.00)	36.62 (51.34)	<0.0001	49.64 (101.00)	35.54 (51.06)	<0.0001
AST	Mean (S.D)	28.30 (23.56)	27.71 (21.50)	0.2974	28.30 (23.56)	27.50 (14.93)	0.1007
HGB	Abnormal	16.09	5.07	<0.0001	16.09	5.81	<0.0001
	Normal	83.91	94.93		83.91	94.19	

CCI, Charlson Comorbidity Index; BMI, Body Mass Index; BP, Blood Pressure; SD, Standard Deviation; BG, Blood Glucose; GTP, Gamma-Glutamyl Transpeptidase; AST, Aspartate Aminotransferase; HGB, Hemoglobin. Abnormal HGB level for men was < 10, and for women was < 12. Variables used in propensity score matching: sex, age, health insurance premium, and CCI.

Variables that were not used in the matching process which were clinical and health behavioral aspects was also showed in Table 1. Analysis of body mass index of the samples revealed higher proportion of underweight individuals among persons with disabilities, while those without disabilities showed higher proportions of overweight individuals. Waist circumferences measures showed no differences between the two groups. Blood pressure measures (both diastolic and systolic), were higher among people without disability, while fasting glucose was higher in those with disability. Kidney function indicators including urine protein and serum creatinine concentrations, were significantly higher (indicating poor kidney function) in persons with disabilities. Similarly, liver function indicators including AST and *γ*-GTP concentrations, were all significantly higher (indicating poorer condition) in persons with disability, even after matching. Regarding health behaviors, persons with disabilities showed higher proportions of non-smokers and non-drinkers, while those without disabilities reported higher levels of physical activity. Notably, the proportion of individuals with low blood hemoglobin concentration (suspicious for disease), was approximately three times higher among those with disabilities even after matching for gender, age, CCI, and health insurance premium.

Table 2 presents the results of multi-level analysis including regional-level variables. (Logistic regression results with individual-level variables only are included in the Supplementary Table 2). Analysis of the entire sample (*n* = 6,788) showed those who were over 70 years of age, women, and obese were more likely to have abnormal hemoglobin level. Regarding health behaviors, physical inactivity was associated with higher risk of abnormal hemoglobin, while alcohol consumption and current tobacco smoking were associated with lower risk. Clinical indicators showed that higher concentrations of creatinine, urine protein, and AST were associated with increased possibilities of abnormal hemoglobin. Among regional variables, the social welfare budget showed a significant correlation with individual hemoglobin concentration, and those living in areas with low welfare budgets were more likely to have abnormal hemoglobin.

**Table 2:** Results of multi-level analysis—associated factors of blood hemoglobin level.

		Abnormal HGB								
		All			w/ disability			w/o disability		
		OR	95% CI	*p*-value	OR	95% CI	*p*-value	OR	95% CI	*p-*value
Individual-level factors										
Sex (ref. male)	Female	0.343	(0.264, 0.445)	<0.0001	0.247	(0.163, 0.374)	<0.0001	0.444	(0.313, 0.629)	<0.0001
Age (ref. 40s)	50–59	1.489	(0.758, 2.924)	0.2481	1.361	(0.575, 3.222)	0.4832	1.852	(0.523, 6.560)	0.3399
	60–69	1.631	(0.840, 3.165)	0.1483	1.012	(0.426, 2.405)	0.9783	3.321	(0.984, 11.205)	0.0531
	70+	3.249	(1.695, 6.230)	0.0004	1.795	(0.770, 4.185)	0.1754	6.515	(1.953, 21.732)	0.0023
Health insurance premium (ref. Q4)	Medical aid	1.424	(0.890, 2.278)	0.1405	0.928	(0.439, 1.964)	0.8456	1.995	(1.067, 3.729)	0.0306
	Q1	0.907	(0.672, 1.225)	0.5256	0.546	(0.330, 0.902)	0.0183	1.223	(0.834, 1.795)	0.3022
	Q2	0.774	(0.565, 1.060)	0.1100	0.619	(0.377, 1.016)	0.0579	0.877	(0.575, 1.337)	0.5426
	Q3	1.089	(0.845, 1.405)	0.5093	0.858	(0.571, 1.289)	0.4614	1.272	(0.907, 1.783)	0.1636
CCI (ref. 0)	1	1.234	(0.947, 1.607)	0.1201	1.401	(0.900, 2.180)	0.1354	1.149	(0.817, 1.615)	0.4255
	2+	1.071	(0.852, 1.345)	0.5574	1.204	(0.839, 1.729)	0.3141	0.976	(0.718, 1.325)	0.8742
BMI (ref. 18.5–23)	< 18.5	1.166	(0.796, 1.708)	0.0004	0.697	(0.389, 1.249)	0.2249	1.968	(1.171, 3.308)	0.0106
	23–25	0.670	(0.509, 0.883)	0.4292	0.800	(0.500, 1.280)	0.3528	0.614	(0.431, 0.874)	0.0067
	> 25	0.479	(0.344, 0.666)	0.0044	0.587	(0.353, 0.976)	0.0400	0.435	(0.277, 0.681)	0.0003
Waist Circum.		0.988	(0.973, 1.003)	0.1300	0.973	(0.951, 0.996)	0.0223	0.999	(0.978, 1.021)	0.9456
Physical activity (ref. ≥3 a week)	None	1.461	(1.159, 1.842)	0.0013	1.593	(1.070, 2.371)	0.0218	1.377	(1.025, 1.851)	0.0340
	< 3 a week	1.373	(0.947, 1.990)	0.0943	1.870	(1.007, 3.474)	0.0474	1.109	(0.678, 1.814)	0.6800
Drinking (ref. non-drinker)	< 3 a month	0.706	(0.444, 1.121)	0.1402	0.657	(0.298, 1.448)	0.2972	0.781	(0.431, 1.415)	0.4148
	1–2 a week	0.550	(0.374, 0.809)	0.0024	0.515	(0.268, 0.989)	0.0463	0.618	(0.375, 1.017)	0.0585
	> 3 a week	0.617	(0.420, 0.906)	0.0138	0.648	(0.331, 1.268)	0.2051	0.659	(0.404, 1.076)	0.0954
Smoking (ref. non-smoker)	Past smoker	0.871	(0.680, 1.114)	0.2707	0.876	(0.585, 1.313)	0.5222	0.843	(0.610, 1.165)	0.3015
	Current smoker	0.602	(0.374, 0.969)	0.0366	0.723	(0.366, 1.429)	0.3510	0.453	(0.215, 0.953)	0.0370
Creatinine		3.759	(2.979, 4.743)	<0.0001	2.810	(2.095, 3.770)	<0.0001	5.903	(4.039, 8.627)	<0.0001
Urine protein		1.136	(1.004, 1.285)	0.0433	1.180	(0.992, 1.404)	0.0611	1.137	(0.952, 1.359)	0.1561
AST		1.010	(1.003, 1.018)	0.0047	1.011	(1.002, 1.020)	0.0125	1.005	(0.990, 1.020)	0.5176
γ-GTP		1.001	(1.000, 1.003)	0.1566	1.000	(0.999, 1.002)	0.6147	1.002	(0.999, 1.005)	0.2948
Disability (ref. no)	Yes	2.218	(1.798, 2.736)	<0.0001						
Regional-level healthcare resources										
No. of doctors per 1,000 (ref. Q4)	Q1	1.015	(0.707, 1.456)	0.9355	0.997	(0.543, 1.833)	0.9930	1.008	(0.621, 1.637)	0.9729
	Q2	1.121	(0.824, 1.525)	0.4675	0.779	(0.445, 1.328)	0.3460	1.405	(0.950, 2.079)	0.0889
	Q3	1.095	(0.814, 1.474)	0.5469	0.806	(0.479, 1.357)	0.4178	1.351	(0.922, 1.978)	0.1225
No. of social facilities per 100,000 (ref. Q4)	Q1	1.343	(0.926, 1.948)	0.1204	1.283	(0.671, 2.455)	0.4513	1.328	(0.820, 2.153)	0.2489
	Q2	1.150	(0.833, 1.587)	0.3954	1.026	(0.591, 1.782)	0.9272	1.259	(0.823, 1.924)	0.2880
	Q3	0.833	(0.600, 1.155)	0.2730	0.881	(0.514, 1.512)	0.6459	0.801	(0.514, 1.248)	0.3269
Social welfare budget (ref. Q4)	Q1	1.594	(1.070, 2.376)	0.0219	2.201	(1.021, 4.000)	0.0434	1.312	(0.773, 2.227)	0.3135
	Q2	1.374	(0.970, 1.946)	0.0735	1.559	(0.841, 2.890)	0.1587	1.299	(0.833, 2.026)	0.2489
	Q3	1.367	(1.028, 1.818)	0.0317	1.503	(0.903, 2.503)	0.1171	1.278	(0.890, 1.836)	0.1840
AIC		2988.63			1168.49			1833.72		
−2 Log L		2908.63			1088.49			1755.72		

HGB, Hemoglobin; OR, Odds Ratio; CI, Confidence Interval; CCI, Charlson Comorbidity Index,; BMI, Body Mass Index; AST, Aspartate Aminotransferase; γ-GTP, Gamma-Glutamyl Transpeptidase; AIC, Akaike Information Criterion. Health premium were categorized with 5 groups: medical aid group and quartiles (Q1–Q4), and 4th quartile is the richest. Variables used in propensity score matching: sex, age, health insurance premium, and CCI. Adjusted variables: diastolic blood pressure, systolic blood pressure, fasting glucose. Abnormal HGB level for men was < 10, and for women was < 12.

Analysis of participants with disabilities (*n* = 1,697) showed that women, individuals with low health insurance premiums, and those who were obese or had wider waist circumferences were less likely to have abnormal hemoglobin levels. Physical inactivity was associated with higher likelihood of abnormal hemoglobin, while alcohol consumption 1–2 times per week was associated with lower likelihood. Higher blood creatinine and AST concentrations, indicating kidney and liver damage respectively, were associated abnormal hemoglobin concentrations. Social welfare budget at the regional-level also showed significant correlation with individual hemoglobin concentration, with areas having the lowest budgets [Q1] showing more than twice the likelihood of abnormal hemoglobin compared to the highest budget areas [Q4].

For participants without disabilities (*n* = 5,091), age was found as the key variable, with those aged 70 years or older being more than six times more likely to have abnormal hemoglobin concentrations. Both BMI and waist circumference were also significant variables, with underweight status increasing the probability of abnormal hemoglobin and obesity decreasing it. In terms of health behaviors, abnormal hemoglobin was higher in people who do not practice physical activity, and it was lower in people who currently smoke tobacco. Blood creatinine and AST concentrations were consistently associated with the likelihood of having abnormal hemoglobin concentrations. Notably, no regional variables showed significant associations in this group.

## 4 Discussion

This study investigated hemoglobin concentration status in Korean populations with and without disabilities by analyzing nationwide claims and check-up data. After 1:3 matching using gender, age, health insurance premium, and CCI, persons with disabilities showed more than twice the proportion of low body weight (BMI less than 18.5) and higher rates of smoking, drinking, and physical inactivity. The prevalence of abnormal hemoglobin levels was approximately three times higher in persons with disability. Additionally, larger standard deviations in clinical variables, including hemoglobin levels, were observed in persons with disabilities, indicating greater variation within this group. This study is the first study to emphasize the health problems focusing on anemia, which can be prevented through community-based nutritional interventions.

The results indicated that men in both groups were more likely to have abnormal hemoglobin levels. While previous studies and national policies have primarily focused on women, particularly adolescent girls and pregnant women, as vulnerable groups, this study revealed that men over 40 years of age, including the elderly may also constitute a vulnerable population regarding low hemoglobin levels. These findings may partially reflect the different criteria for abnormal hemoglobin in men (<12 g/dL) and women (<10 g/dL). Although women generally face stronger association of anemia, some studies demonstrate increased anemia incidence in elderly men with age (40, 41). The study’s focus on middle-aged and elderly people over 40 years of age, including the elderly, along with the matched sample of persons with disabilities, included a relatively large proportion of older individuals, potentially influencing these results (mean ages before matching: 67.02 years for those with disabilities, 59.87 years for those without).

Age emerged as a significant factor only in the sample of those without disability. Individuals aged 70 years or older were significantly more likely to have abnormal hemoglobin levels. Using health insurance premiums as a proxy for economic status, contrasting patterns emerged. Among persons with disability, those with relatively high income showed higher likelihood of abnormal hemoglobin, while among people with no disability, those with low income were more likely to have abnormal hemoglobin. This inverse relationship among persons with disability may be attributed to existing support system for low-income disabled population, enabling service access and usage within the system.

Regarding health behaviors, both groups showed similar patterns. Physical inactivity was associated with higher probability of abnormal hemoglobin, while moderate smoking or drinking was associated with lower probability of abnormal hemoglobin. These findings suggest that individuals that can engage in smoking and drinking may be able to do so because their current health condition is relatively good (42–44).

Furthermore, kidney and liver function showed significant associations with blood hemoglobin levels in both groups. In particular, persons with disability who also have chronic kidney disease face heightened risk of developing additional health problems due to anemia, emphasizing the need for continued research and treatment methods to manage their hemoglobin levels.

Among regional health and welfare resource variables, the ‘ratio of social welfare budget’ was significantly associated to individual hemoglobin concentration. This finding demonstrates the substantial impact of community resources on the health management for persons with disabilities. The social welfare budget ratio reflects population size, distribution of vulnerable populations, and financial status, and is reported to vary by region (45). While this indicator does not directly measure budget allocation for nutrition projects for persons with disability, it suggests that regions with higher welfare budgets may have implemented projects that have benefited the nutritional problems of the vulnerable populations. In fact, Korean nutritional support programs typically focus on direct food provision to participants (46). While national health promotion programs under the Ministry of Health and Welfare, such as the Nutrition Plus Program and Customized Nutrition Management Program, include nutrition counseling services, locally run programs primarily utilize food provision methods, including meal services through welfare facilities and meal delivery.

This study offers several methodological contributions. First, this study compared and analyzed factors affecting the hemoglobin levels of people with and without disability by matching their characteristics through propensity score matching (PSM). Because while claims data analysis uses nationally representative data, it cannot replicate the effects of clinical research such as randomized controlled trials (RCTs), PSM helped to minimize group differences. Although PSM may retain hidden bias due to limitation of included variables, it helped to address the limitations of claims data analysis by maximizing similarity of the two groups.

This study also analyzed claims data from the entire population, targeting individuals who registered their disabilities during 2010–2019 and their matched non-disabled counterparts. Unlike previous studies primarily focusing on persons with congenital disabilities, our database enabled the identification of disability registration timing through claims data. The utilization of nationwide health insurance claims database, representing both populations with and without disability, further strengthens this study. Previous studies on the persons with disability in Korea had limitations in that they conducted with only for some people with specific disabilities were limited by their focus on specific disability types or reliance on subjective questionnaires. In contrast, this study conducted comprehensive analysis including all disabled people for whom screening data were available. However, this study analyzed the disabled population as a single group, and the diversity within the disabled population was not considered, which may need to be addressed in future research.

The political implication of this study is as follows. Through multi-level analyses, we identified regional variations in the blood hemoglobin concentration among individuals with disabilities, specifically finding that social welfare resources, rather than healthcare resources, significantly impact nutritional problems in persons with disabilities. Based on the national survey (19), persons with disabilities are known to be more vulnerable than non-disabled persons in terms of chronic diseases, education, and income level, and therefore, the social welfare budget for supporting the health and nutrition of vulnerable population is interpreted to have a more significant impact on persons with disabilities than on non-disabled persons.

In South Korea, healthcare for the persons with disabilities emphasizes social welfare rather than medical care. While ‘The Comprehensive Health Plan for the People with Disability’, scheduled for implementation in 2024, seeks to include plans to expand the primary care physician system, it lacks direct nutrition-related initiatives (36). Although a ‘Nutritional management for the persons with disability’ program exists, it operates only in limited areas (47). Given the findings of this study that showed persons with disabilities are more likely to have nutritional problems, such as abnormal hemoglobin levels, it is imperative to implement more comprehensive nutrition aid projects. These projects should focus on improving blood hemoglobin levels and providing targeted screening program for the persons with disabilities. This study provides foundational evidence for establishing nutrition and health-related agendas for persons with disabilities. Implementation of policies focusing on awareness improvement and nutrition projects is also necessary.

Despite the implications, this study yields some limitations. First, the analysis’s restriction to 2018–2019 data precluded long-term follow-up and determination of causal relationships between factors and hemoglobin concentration. Additionally, there may potential selection bias. As the study utilized claims check-up data, it may oversample employed individuals and those who are able to receive a medical examination. Among persons with disability, those with mobility challenges, those who without a caregiver, and those with severe disabilities may been excluded from the analysis. Therefore, it is possible that the burden of anemia among persons with disabilities may be underestimated in this study. Lastly, due to limitation of claims data, this analysis did not consider diet and nutrient intake, which is the major contributor to blood hemoglobin concentration.

Based on these considerations, several areas for future research are recommended. Given that this study examined the population with disabilities as a single group, analysis of differences according to disability characteristics, such as mental vs. physical disabilities, would be valuable. Furthermore, future studies are needed that consider factors affecting hemoglobin levels that were not considered in this study, and it would also be beneficial to conduct studies that track additional health outcomes beyond hemoglobin levels according to disability.

## 5 Conclusion

This study investigated individual and regional-level factors associated with low hemoglobin levels in people with and without disabilities using a nationwide health insurance claims database with check-up data. PSM analysis identified gender, BMI, health behavior variables, and kidney and liver functions as significant factors in both groups. Notably, disability was significantly associated with lower hemoglobin level, even after matching. Analysis of persons with disability revealed that the level of local welfare resources was a significant factor, confirming the important role of community welfare and medical resources in improving the nutritional status and overall health of persons with disability. The findings and implications of this study could be further developed through future research that considers disability type and duration, and enables follow-up investigation of health outcomes.

## Data Availability

The data analyzed in this study is subject to the following licenses/restrictions: please state the restrictions that apply to the dataset. Requests to access these datasets should be directed to nhiss.nhis.or.kr/.
