# Supplementary figures and images for: Status and factors related to hemoglobin concentration of people with vs. without disability—using nationwide claims check-up database
Source: Front Nutr. 2025 Mar 19;12:1519098. doi: 10.3389/fnut.2025.1519098 (PMC11963805; doi:10.3389/fnut.2025.1519098)

Supplementary figure 1. The distribution of propensity score before and after matching

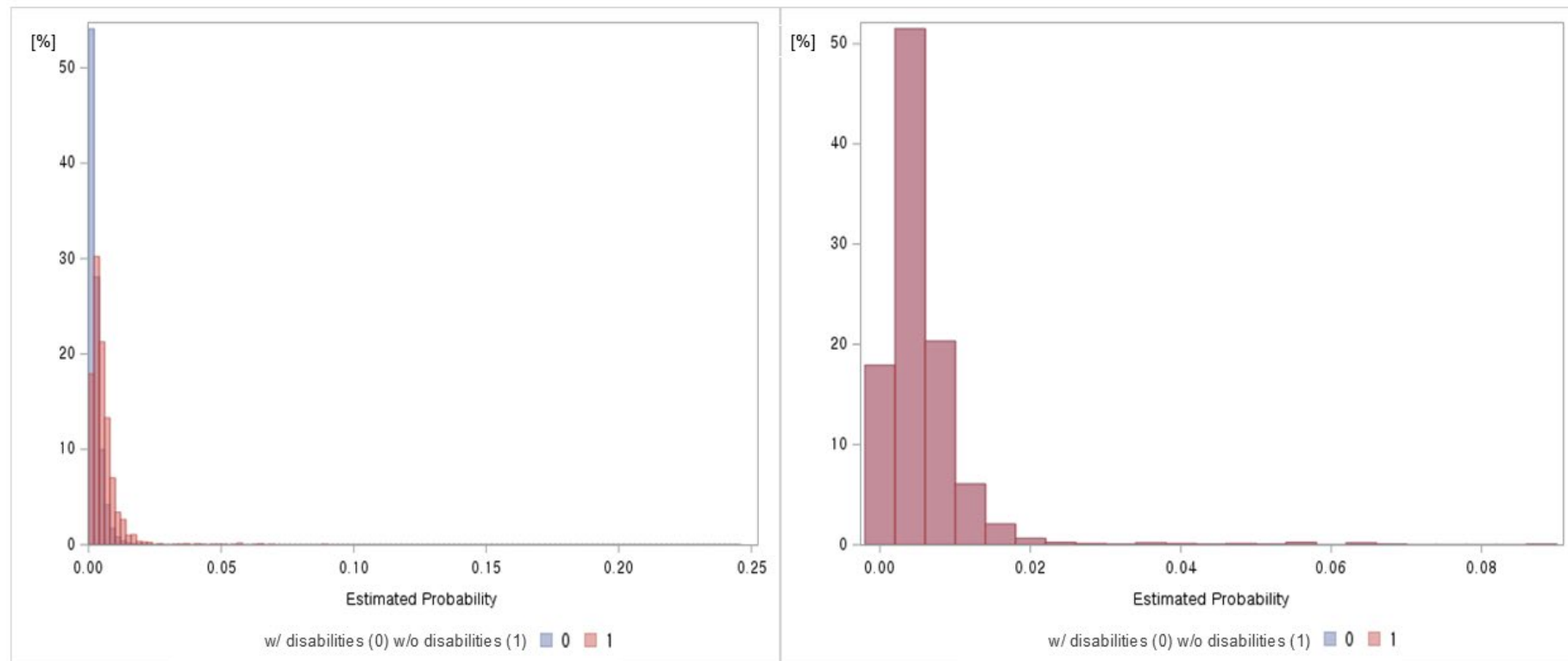

Supplement: Supplementary file 1 [file Image_1.pdf]
