# Supplementary material for: Status and factors related to hemoglobin concentration of people with vs. without disability—using nationwide claims check-up database
Source: Front Nutr. 2025 Mar 19;12:1519098. doi: 10.3389/fnut.2025.1519098 (PMC11963805; doi:10.3389/fnut.2025.1519098)
Supplement: Supplementary file 2 [file Table_1.docx]

Supplementary table 1. Description of regional healthcare resources variables included in the model

| N=252 | | | |
| --- | --- | --- | --- |
|  | Mean | SD | CV |
| No. of doctors per 1,000 people in 2018 | 2.72 | 2.31 | 0.85 |
| Q1 | 1.52 | 0.19 |  |
| Q2 | 1.96 | 0.11 |  |
| Q3 | 2.44 | 0.20 |  |
| Q4 | 4.94 | 3.76 |  |
| No. of social welfare facilities per 100,000 people in 2018 | 18.80 | 11.69 | 0.62 |
| Q1 | 7.06 | 2.00 |  |
| Q2 | 13.22 | 2.14 |  |
| Q3 | 20.10 | 2.40 |  |
| Q4 | 34.99 | 9.94 |  |
| Ratio of social welfare budget in 2018 | 33.65 | 14.20 | 0.42 |
| Q1 | 17.90 | 1.96 |  |
| Q2 | 25.10 | 2.84 |  |
| Q3 | 37.11 | 3.77 |  |
| Q4 | 54.60 | 5.24 |  |

SD: Standard Deviation, CV: Coefficient of Variation
