# Supplementary material for: Status and factors related to hemoglobin concentration of people with vs. without disability—using nationwide claims check-up database
Source: Front Nutr. 2025 Mar 19;12:1519098. doi: 10.3389/fnut.2025.1519098 (PMC11963805; doi:10.3389/fnut.2025.1519098)
Supplement: Supplementary file 3 [file Table_2.docx]

Supplementary table 2. Associated factors of blood hemoglobin level – individual level only

|  | | **Abnormal HGB** | | | | | | | | | | | |
| --- | --- | --- | --- | --- | --- | --- | --- | --- | --- | --- | --- | --- | --- |
|  |  | **All** | | | | **w/ disability** | | | | **w/o disability** | | | |
|  |  | OR | CI | | p | OR | CI | | p | OR | CI | | p |
| Disability (ref. no) | yes | 2.222 | 1.804 | 2.736 | <.0001 | - | - | - | - | - | - | - | - |
| Sex (ref. male) | female | 0.344 | 0.266 | 0.447 | <.0001 | 0.260 | 0.174 | 0.390 | <.0001 | 0.438 | 0.310 | 0.619 | <.0001 |
| Age (ref. 40s) | 50-59 | 1.512 | 0.771 | 2.967 | 0.229 | 1.491 | 0.643 | 3.459 | 0.352 | 1.882 | 0.532 | 6.660 | 0.327 |
|  | 60-69 | 1.642 | 0.847 | 3.183 | 0.142 | 1.097 | 0.472 | 2.551 | 0.830 | 3.333 | 0.989 | 11.230 | 0.052 |
|  | 70+ | 3.320 | 1.736 | 6.351 | 0.000 | 1.980 | 0.868 | 4.518 | 0.105 | 6.534 | 1.965 | 21.725 | 0.002 |
| Health premium  (ref. Q4) | aid | 1.414 | 0.886 | 2.256 | 0.146 | 1.007 | 0.488 | 2.074 | 0.986 | 1.894 | 1.017 | 3.529 | 0.044 |
|  | Q1 | 0.891 | 0.661 | 1.201 | 0.448 | 0.539 | 0.331 | 0.879 | 0.013 | 1.208 | 0.826 | 1.766 | 0.329 |
|  | Q2 | 0.767 | 0.561 | 1.049 | 0.097 | 0.610 | 0.377 | 0.987 | 0.044 | 0.870 | 0.572 | 1.323 | 0.515 |
|  | Q3 | 1.074 | 0.833 | 1.383 | 0.582 | 0.832 | 0.561 | 1.232 | 0.358 | 1.255 | 0.897 | 1.757 | 0.184 |
| CCI (ref. 0) | 1 | 1.233 | 0.947 | 1.604 | 0.119 | 1.437 | 0.938 | 2.203 | 0.096 | 1.147 | 0.818 | 1.608 | 0.427 |
|  | 2+ | 1.082 | 0.863 | 1.357 | 0.495 | 1.262 | 0.893 | 1.784 | 0.187 | 0.992 | 0.732 | 1.345 | 0.958 |
| BMI  (ref. 18.5-23) | <18.5 | 1.188 | 0.813 | 1.736 | 0.373 | 0.710 | 0.404 | 1.247 | 0.233 | 2.010 | 1.204 | 3.357 | 0.008 |
|  | 23-25 | 0.670 | 0.509 | 0.881 | 0.004 | 0.813 | 0.515 | 1.282 | 0.372 | 0.611 | 0.430 | 0.870 | 0.006 |
|  | > 25 | 0.476 | 0.342 | 0.661 | <.0001 | 0.579 | 0.353 | 0.949 | 0.030 | 0.441 | 0.282 | 0.689 | 0.000 |
| Waist Circumference |  | 0.988 | 0.973 | 1.003 | 0.125 | 0.973 | 0.951 | 0.995 | 0.018 | 0.999 | 0.978 | 1.020 | 0.936 |
| Physical activity  (ref. ≥3 a wk.) | none | 1.479 | 1.175 | 1.862 | 0.001 | 1.602 | 1.090 | 2.356 | 0.017 | 1.409 | 1.052 | 1.888 | 0.022 |
|  | <3 a week | 1.386 | 0.958 | 2.007 | 0.083 | 1.983 | 1.088 | 3.613 | 0.025 | 1.114 | 0.683 | 1.819 | 0.665 |
| Drinking  (ref. non-drinker) | <3 a month | 0.701 | 0.442 | 1.110 | 0.130 | 0.641 | 0.298 | 1.376 | 0.253 | 0.779 | 0.432 | 1.407 | 0.408 |
|  | 1-2 a week | 0.549 | 0.374 | 0.806 | 0.002 | 0.489 | 0.259 | 0.924 | 0.028 | 0.610 | 0.372 | 1.001 | 0.050 |
|  | >3 a week | 0.634 | 0.432 | 0.929 | 0.019 | 0.712 | 0.375 | 1.351 | 0.298 | 0.666 | 0.409 | 1.084 | 0.102 |
| Smoking  (ref. non-smoker) | past smoker | 0.863 | 0.676 | 1.102 | 0.238 | 0.881 | 0.597 | 1.302 | 0.526 | 0.833 | 0.605 | 1.149 | 0.266 |
|  | currently | 0.600 | 0.374 | 0.964 | 0.035 | 0.717 | 0.373 | 1.379 | 0.319 | 0.446 | 0.212 | 0.936 | 0.033 |
| Blood Creatinine |  | 3.737 | 2.967 | 4.706 | <.0001 | 2.935 | 2.198 | 3.920 | <.0001 | 5.686 | 3.910 | 8.268 | <.0001 |
| Urine protein |  | 1.130 | 1.000 | 1.277 | 0.050 | 1.140 | 0.965 | 1.346 | 0.122 | 1.142 | 0.958 | 1.361 | 0.138 |
| AST |  | 1.010 | 1.003 | 1.017 | 0.006 | 1.010 | 1.002 | 1.018 | 0.018 | 1.005 | 0.991 | 1.020 | 0.487 |
| γ-GTP |  | 1.001 | 1.000 | 1.003 | 0.147 | 1.000 | 0.999 | 1.002 | 0.592 | 1.002 | 0.999 | 1.005 | 0.307 |
| AIC |  | 2982.05 | | | | 1158.53 | | | | 1827.05 | | | |
| -2 Log L |  | 2920.05 | | | | 1098.53 | | | | 1767.05 | | | |

CCI: Charlson Comorbidity Index, BMI: Body Mass Index, AST: Aspartate Aminotransferase, γ-GTP: Gamma-glutamyl Transpeptidase, AIC: Akaike Information Criterion

Variables used in propensity score matching: sex, age, health insurance premium, and CCI

Adjusted variables: diastolic blood pressure, systolic blood pressure, fasting glucose

Abnormal HGB level for men was <10, and for women was <12.
